# A Preliminary Study on Elimination of Colonies of the Mound Building Termite *Macrotermes gilvus* (Hagen) Using a Chlorfluazuron Termite Bait in the Philippines

**DOI:** 10.3390/insects2040486

**Published:** 2011-11-10

**Authors:** Partho Dhang

**Affiliations:** Independent Consultant 2410, Hen Belarmino Street, Bangkal, Makati City 1233, Philippines; E-Mail: partho@urbanentomology.com; Tel.: +63-2-845-1838

**Keywords:** *Macrotermes gilvus*, chlorfluazuron, termite baiting

## Abstract

The effectiveness of a chlorfluazuron termite bait in eliminating colonies of the termite species *Macrotermes gilvus* (Hagen) was evaluated under field conditions. Three active termite mounds were chosen for this study, two acted as test mounds and the other as the control. Four In-Ground Stations (IGS) were installed around each mound. Interception occurred almost immediately in all the stations, which were subsequently baited. The control mound was fed a bait matrix lacking the active ingredient. Stations were re-baited every 2 weeks for 10–12 weeks until bait consumption ceased in the test mounds. The mounds were left undisturbed for four more weeks before being destructively sampled. The desiccated remains of workers, soldiers, late instars and queen were found upon sampling the treated mounds. A few live termites were located in one treated mound but were darkly pigmented indicating bait consumption. The control mound remained healthy and did not show any visible sign of negative impact. The bait successfully suppressed or eliminated both *M. gilvus* colonies within 16 weeks from commencement of feeding.

## 1. Introduction

Termite control is estimated to be the largest segment in the global pest control industry and worth 8 billion US dollar [1]. The economic damage caused by termites amounts to billions of dollars in the United States, Australia and Japan, but never has been quantified in developing countries.Termite control has been further aggravated by the withdrawal of the persistent organochlorines as soil barrier treatments from world markets. The replacement termiticides now available are less persistent, thus placing structures under a more rigorous inspection regime and likely requiring repeated treatments.

Termite baiting systems have recently evolved as a major technique to protect structures and timber against termites. This technique makes use of the inherent termite behaviors of interdependence, trophallaxis, mutual grooming and cannibalism to distribute the bait toxicant throughout the colony, resulting in population loss and sometimes colony elimination. A number of bait toxicants and baiting systems have been developed and evaluated over the last few decades for the control of subterranean termite species. Successfully commercialized among them are hexaflumuron [2], diflubenzuron [3], chlorfluazuron [4,5], and noviflumuron [6]. Much of the published data has however been restricted to tests on invasive termite species belonging to the genera *Coptotermes or Reticulitermes*. There is limited published data regarding termite bait efficacy against other genera particularly those belonging to higher group of termites. One recent study however has proven the success of baiting in eliminating higher termite *Globitermes sulphureus* [7].

The current study investigates the efficacy of a chlorfluazuron-termite bait on active *Macrotermes gilvus* termite mounds. The species is commonly known as the Philippine mound building termite and belongs to the order Termitidae. It is generally found in rural and forested areas of the country. However, various local pest control forums and conferences have lately categorized this species as an urban and suburban pest of structures needing control measures. Chlorfluazuron is a benzoylphenyl urea that acts as a potent chitin synthesis inhibitor, across various orders of insects [8,9]. Ingestion of chitin synthesis inhibitor results in abnormal deposition of chitin and abortive molting.

## 2. Experimental Section

### 2.1. Materials

All materials used for the study were provided by Ensystex Inc., Fayettevile, NC, USA. These include In-Ground Stations (IGS), termite bait (containing 1.0 g chlorfluazuron/kg of bait matrix, control bait (bait matrix without chlorfluazuron), timber interceptors made from *Eucalyptus regnans*, a non native species of hardwood and various specialist tools.

### 2.2. Site Description

The trial was established on a property in Antipolo, 55 km north from Manila City, Philippines. The one-hectare of suburban property was covered with fruit trees and had more than 15 *M. gilvus* mounds of various sizes. The work was conducted between October and December, months which are generally cooler with fair precipitation.

### 2.3. Mound Selection

This study was designed to determine the effect of baiting on termites by using active *M. gilvus* mounds. For this purpose three active mounds were chosen, that were not less than 50 meters from each other. Two mounds acted as a treatment and one as a control mound.

### 2.4. Installation of IGS and Baiting

Based on prior observations by the author on station interception and rate of feeding, four IGS, each with 6 timber interceptors, were placed around each of the three mounds. The distance of the IGS was fixed approximately 100 cm from the center of each mound. The IGS were locked and kept undisturbed for a period of 2 weeks before the first inspection. Once it was determined that feeding on the interceptors had occurred, the termite bait was added. The control mound received the bait lacking the active ingredient.

### 2.5. Bait Preparation and Application

The termite bait is in the form of processed cellulose powder consisting of proprietary cellulose-feeding matrix (999.0 g/Kg of the final bait matrix) with the bait toxicant chlorfluazuron (1.0 g/Kg) [5]. To prepare the bait, distilled water was added to the powder in a clean plastic bucket and the mixture stirred using a spatula to achieve a “dough” type consistency. A mixing ratio of 250 g of bait to 1000 mL of water was used. A plastic scoop was used to fill the IGS with the bait mixture. The amount of dry bait in grams was measured before adding it to IGS throughout the study period.

### 2.6. Monitoring Colony Vigor

Colony vigor was determined by inspecting the IGS every two-week. Stations were replenished with fresh bait as required based on consumption. The monitoring and bait supplementation process continued until a complete cessation of feeding activity was noticed in the test mounds. However the control mound continued to receive the control bait until destruction.

### 2.7. Mound Destruction

On cessation of feeding in the test mounds, a 4 week time interval was strictly followed prior to mound destruction. During this 4 week period IGS with uneaten bait were kept undisturbed, allowing the termites to return and initiate feeding. After the 4 weeks from cessation of feeding the mounds were destructively sampled and the soil thoroughly examined for termites. The control mound was also sampled similarly.

## 3. Results and Discussion

### 3.1. First Inspection

The site was inspected two weeks after installation of the IGS on the two test and one control mounds. Termites were found consuming the wooden interceptors in all the stations. This indicated that all three mounds selected were active and fit for continuation of the test.

### 3.2. Baiting and Subsequent Inspections

#### Week 2

Following successful interception of termites in the IGS, the termite bait was added to each station. In the control mound the control bait as added. The bait mixture in the form of “dough” was loosely dropped in the station without compacting until the stations were completely filled.

#### Week 4 and 6

Inspection revealed large numbers of worker termites and near total consumption of bait in most stations. A large amount of mud was observed in all the stations and filled the space previously occupied by the bait. The degree of consumption varied but not visually different among all the IGS. Most bait in the IGS visually showed signs of over 90 percent consumption and no station similarly showed less than 60 percent consumption. Mostly worker termites were found feeding in the stations. Regardless of feeding, all stations were replenished with bait to allow continuous feeding.

#### Weeks 8

A considerable reduction in feeding was noticed from week 8 onwards in all treated mounds. All the IGS on the test mounds from week 8 onwards showed a large amount of uneaten bait with lesser numbers of active termites compared to all previous visits. The ratio of soldiers to workers in the stations showed a marked increase compared to that previously noticed.

#### Week 10 and 12

A complete cessation in bait feeding was observed at week 10 for mound 1 and week 12 for test mound number 2. The total amount of bait used through week 12 is depicted in Table 1. The stations were left undisturbed with the uneaten bait for 4 weeks for further monitoring.

#### Week 16

A final inspection to confirm colony elimination occurred 16 weeks from the date of installation of IGS on the test mounds. These mounds were thus identified as potentially eliminated due to the lack of visible activity and absence of repair works on inspection spots. Each mound was destructively sampled using a pick and shovel and observations made. The control mound was sampled simultaneously.

Careful observation of the content of the test mounds revealed a lack of termite activity inside. Further examination revealed desiccated bodies of worker and soldier castes in a number of spots inside the mounds. The dead termites were bundled together in mass. No live workers were found in the test mound 1. In test mound 2, a few primary and secondary soldiers were found. A few early instars were located in the mound also. These were possibly eggs when the bait was being consumed and hatched during the course of colony elimination.

The queen and king could not be located in the royal chamber in mounds 1, but in mound 2 a dead queen was located in the royal chamber with a few live worker and soldier termites in the immediate vicinity. The bodies of these workers showed dark pigmentation characteristic with bait consumption.

The control mound showed no visible effects and termites continued to feed on the control bait until destroyed. The weekly consumption of the bait remained high and uniform during this period.

## 4. Conclusions

This study proved that a termite bait using chlorfluazuron as an active ingredient may suppress and possibly eliminate colonies of *M. gilvus*. The exact mode of transfer of the bait toxicant to the colony is unclear in this preliminary study and efforts are being made to determine this in another study.

## Figures and Tables

**Table 1:** Total amount of termite bait (dry weight) added for each of the sampled *M. gilvus* mounds.

Duration	Amount of bait added per mound (dry weight)		
	Control Mound	Treated Mound 1	Treated Mound 2
Week 2	1000 gm	1000 gm	1000 gm
Week 4	800 gm	800 gm	800 gm
Week 6	800 gm	600 gm	600 gm
Week 8	800 gm	200 gm	600 gm
Week 10	750 gm	0 gm	150 gm
Week 12	800 gm	0 gm	0 gm
Total week 12	4950 gm	2600 gm	3150 gm
